# Corticosteroid-induced mania, review and case report

**DOI:** 10.1192/j.eurpsy.2024.900

**Published:** 2024-08-27

**Authors:** M. García Moreno, A. De Cos Milas, L. Beatobe Carreño, A. Izquierdo De La Puente, P. Del Sol Calderon

**Affiliations:** ^1^Psychiatry, Hospital Universitario Puerta de Hierro Majadahonda; ^2^Psychiatry, Hospital Universitario De MóStoles, Madrid, Spain

## Abstract

**Introduction:**

Corticosteroid treatment has been associated with the appearance of psychiatric symptoms such as depression, mania or psychosis. It is believed that manic symptoms appear with lower doses than psychotic ones. Furthermore, manic symptoms are usually associated with brief treatments against depressive ones that often appear with chronic administration of corticosteroids. The symptoms can persist for to 2 months, with an average duration of 3 weeks. The prognosis is favorable with a complete remission of symptoms in more than 90% of patients. Treatment initially consists in reducing or removing corticosteroids. However, sometimes symptomatic treatment with antipsychotics or mood stabilizers is necessary.

**Objectives:**

To review about corticosteroid-induced mania

**Methods:**

We carry out a literature review about corticosteroid-induced mania, accompanied by a clinical description of one patient previously diagnosed of bipolar disorder who presents a manic episode after corticosteroids treatment.

**Results:**

A 25-year-old male was admitted to the short-term hospitalization unit from the emergency department due to manic symptoms. He had a previous diagnosis of attention deficit hyperactivity disorder sin adolescence and also a diagnosis of bipolar disorder established 7 years ago. During the last year he had received treatment with asenapine 10 mg and lamotrigine 200 mg, with good response. Several weeks before his admission he received corticosteroid treatment during several days, due to an respiratory infection. In this context he appeared more nervous, dysphoric, hyperthymic, impulsive, with increased speech pressure, insomnia and tachypsychia. Despite the withdrawal of corticosteroid treatment, manic symptoms persisted. During admission, asenapine´s dose was increased with a complete remission of the manic symptoms.

**Conclusions:**

Corticosteroids are associated in a high percentage with the appearance of manic symptoms. The prognosis is usually favorable after the withdrawal of corticosteroid treatment. However, sometimes the symptoms do not disappear despite withdrawal - mainly due to individual vulnerability - or this one is not possible. In these cases, treatment with antipsychotics or mood stabilizers is indicated.

**Disclosure of Interest:**

None Declared

